# FNDC5 Attenuates Oxidative Stress and NLRP3 Inflammasome Activation in Vascular Smooth Muscle Cells via Activating the AMPK-SIRT1 Signal Pathway

**DOI:** 10.1155/2020/6384803

**Published:** 2020-05-16

**Authors:** Bing Zhou, Yun Qiu, Nan Wu, Ai-Dong Chen, Hong Zhou, Qi Chen, Yu-Ming Kang, Yue-Hua Li, Guo-Qing Zhu

**Affiliations:** ^1^Key Laboratory of Targeted Intervention of Cardiovascular Disease, Collaborative Innovation Center of Translational Medicine for Cardiovascular Disease, and Department of Physiology, Nanjing Medical University, Nanjing, Jiangsu 211166, China; ^2^Department of Pathophysiology, Nanjing Medical University, Nanjing, Jiangsu 211166, China; ^3^Department of Physiology and Pathophysiology, Cardiovascular Research Center, Xi'an Jiaotong University School of Medicine, Xi'an 710061, China

## Abstract

Vascular oxidative stress and inflammation play a major role in vascular diseases. This study was aimed at determining the protective roles of fibronectin type III domain-containing 5 (FNDC5) in angiotensin II- (Ang II-) induced vascular oxidative stress and inflammation and underlying mechanisms. Wild-type (WT) and FNDC5^−/−^ mice, primary mouse vascular smooth muscle cells (VSMCs), and the rat aortic smooth muscle cell line (A7R5) were used in the present study. Subcutaneous infusion of Ang II caused more serious hypertension, vascular remodeling, oxidative stress, NLRP3 inflammasome activation, AMPK phosphorylation inhibition, and SIRT1 downregulation in the aorta of FNDC5^−/−^ mice than those of WT mice. Exogenous FNDC5 attenuated Ang II-induced superoxide generation, NADPH oxidase 2 (NOX2) and NLRP3 upregulation, mature caspase-1, and interleukin-1*β* (IL-1*β*) production in A7R5 cells. The protective roles of FNDC5 were prevented by SIRT-1 inhibitor EX527, AMPK inhibitor compound C, or integrin receptor inhibitor GLPG0187. FNDC5 attenuated the Ang II-induced inhibition in SIRT1 activity, SIRT1 protein expression, and AMPK*α* phosphorylation in A7R5 cells, which were prevented by compound C, EX527, and GLPG0187. FNDC5 deficiency deteriorated Ang II-induced oxidative stress, NLRP3 inflammasome activation, AMPK phosphorylation inhibition, and SIRT1 downregulation in primary aortic VSMCs of mice, which were prevented by exogenous FNDC5. These results indicate that FNDC5 deficiency aggravates while exogenous FNDC5 alleviates the Ang II-induced vascular oxidative stress and NLRP3 inflammasome activation via the AMPK-SIRT1 signal pathway in VSMCs.

## 1. Introduction

Chronic vascular inflammation greatly contributes to the pathogeneses of hypertension, atherosclerosis, and aortic aneurysm [1–3]. Accumulated studies in animals and humans have revealed a great contribution of inflammation to vascular oxidative stress [4–6]. Anti-inflammation therapies have protective effects in cardiovascular diseases, and normalization of oxidative stress is an essential characteristic of these therapies [7]. Oxidative stress represents excessive intracellular reactive oxygen species (ROS), which promotes inflammation, and greatly assists in the pathogenesis of cardiovascular diseases [8]. The ROS are important oxidative stressors implicated in driving vascular diseases by promoting vascular inflammation, increasing the proliferation, migration, and apoptosis of the vascular smooth muscle cells (VSMCs), and thereby stimulating vascular remodeling [9–11].

Renin-angiotensin system (RAS) plays an important role in the pathogenesis of cardiovascular diseases, and intervention of the RAS plays beneficial effects in cardiovascular diseases [12]. Angiotensin II (Ang II) is a key effector peptide of the RAS, which promotes VSMC proliferation, migration, apoptosis, oxidative stress, and inflammation as well as vascular remodeling [13]. Ang II stimulates the ROS production primarily through nicotinamide adenine dinucleotide phosphate (NADPH) oxidases (NOXs) and induces inflammation which is closely related to the activation of nod-like receptor protein 3 (NLRP3) inflammasome in VSMCs and arteries [14]. NLRP3 inflammasome is a cytosolic protein complex including NLRP3, ASC, and caspase-1 [15]. When the inflammasome is assembled, procaspase-1 turns to its active form caspase-1, which further converts pro-interleukin-1*β* (pro-IL-1*β*) into its active form IL-1*β*, and thus triggers inflammatory responses [16]. The inflammasome activation plays roles in the phenotypic transformation and proliferation of VSMCs in hypertension [17]. ROS serve as a triggering factor to activate NLRP3 inflammasome [18, 19]. Application of antioxidants to scavenge excessive ROS attenuates inflammatory responses via inhibiting NLRP3 inflammasome activation [20–22].

Fibronectin type III domain containing 5 (FNDC5) is a transmembrane protein, and irisin is a peptide from the cleavage of the extracellular domain of FNDC5 [23]. FNDC5 attenuates the disturbance of glucose and lipid metabolism, insulin resistance, and hepatosteatosis [24–26]. It inhibits inflammation in adipose tissues of high-fat diet-induced obesity rats [27] and in adventitial fibroblasts of spontaneously hypertensive rats (SHR) [28]. However, it is still undetermined whether FNDC5 would attenuate oxidative stress and inflammation in VSMCs. This study concentrates on the roles of FNDC5 in Ang II-induced oxidative stress and inflammation and its underlying mechanisms in rat aortic smooth muscle cell line (A7R5), VSMCs, and aortas of wild-type (WT) mice and FNDC5^−/−^ mice.

## 2. Materials and Methods

### 2.1. Animals

Male wild-type (WT) and FNDC5^−/−^ mice on a C57BL/6 background were available from Nanjing Medical University (Nanjing, Jiangsu, China). The experiments conformed to the Guide for the Care and Use of Laboratory Animal (US National Institutes of Health, NIH publication, 8th edition, 2011). The mice were housed in a temperature-controlled room with a 12 h light–dark cycle and free access to standard chow and tap water. The mice were euthanized with an intravenous injection of an overdose of pentobarbital sodium (200 mg/kg) at the end of the experiment.

### 2.2. Cell Culture and Treatment

The rat aortic smooth muscle cell line (A7R5) was obtained from American Type Culture Collection (Manassas, VA, USA). Primary mouse VSMCs were isolated from the thoracic aorta of WT and FNDC5^−/−^ mice aged 8 weeks and cultured as described previously [29]. The VSMCs between the second and sixth passages were used for the present study. The cells with a density at 80-90% were treated with Ang II (100 nM) for 24 h to induce oxidative stress and inflammation in VSMCs [30–34].

### 2.3. Mouse Model of Hypertension

Ang II was utilized to induce hypertension accompanied with vascular remodeling, oxidative stress, and inflammation [35–38], which is a better animal model of human essential hypertension [39]. The WT and FNDC5^−/−^ mice were subjected to subcutaneous infusion of saline or Ang II (400 ng/kg/min for 2 weeks) with an osmotic minipump (ALZET 1002, Durect Corporation, Mountain View, CA, USA) [39]. The blood pressure of tail artery was examined in a conscious state with a noninvasive computerized tail-cuff system (NIBP, AD Instruments, Sydney, Australia). The data were obtained by averaging 10 measurements [40].

### 2.4. Western Blot Analysis

VSMCs or aortic media were homogenized in lysis buffer. The supernatant was extracted, and the total protein was measured. Protein was separated by SDS-PAGE and transferred to PVDF membranes. The bands were visualized with the Enhanced Chemiluminescence Detection Kit (Thermo Scientific, Rockford, IL, USA). Antibodies against NLRP3 (No. ab214185), SIRT1 (No. ab110304), FNDC5 (No. ab174833), and NOX2 (No. ab129068) were purchased from Abcam (Cambridge, MA, USA). Antibodies against p-AMPK (4184S) and *β*-actin (No. 3700S) were acquired from Cell Signaling Technology (Beverly, MA, USA). IL-1*β* antibody (No. sc-12742) and caspase-1 antibody (No. sc-56036) were purchased from Santa Cruz Biotechnology (Santa Cruz, CA, USA). The former detected pro-IL-1*β* at 31 KDa and IL-1*β* at 17 KDa, and the latter showed procaspase-1 at 45 KDa and caspase-1 at 10 KDa. Antibodies against AMPK (No. 10929-2-AP), NOX4 (No. 14347-1-AP), and ASC (No. 10500-1-AP) were purchased from Protein Tech Group Inc. (Chicago, IL, USA).

### 2.5. Masson's Staining

Aortas of mice were prefixed, and the paraffin-embedded sections were stained with Masson's trichrome staining as we previously reported [41, 42]. The images were collected with a light microscope (BX-51, Olympus, Tokyo, Japan). The aortic medium thickness and medium area were used as indexes of vascular remodeling.

### 2.6. DHE Fluorescence Staining

Dihydroethidium (DHE) fluorescence staining was used to evaluate intracellular ROS levels [43, 44]. For VSMCs, cells (3 × 10^5^ cells/mL) were seeded in the six-well plates and incubated with DHE (10 *μ*M) in PBS at 37°C for 30 min in a dark and humidified container and, then, washed twice with cold PBS. For aortas, the sections were embedded in OCT and then incubated with DHE (10 *μ*M) for 5 min at room temperature and rinsed two times with PBS. The fluorescence was detected under excitation at 518 nm and emission at 605 nm with a fluorescence microscopy (DP70, Olympus Optical, Tokyo, Japan).

### 2.7. RT-PCR

Total RNA was exacted with a Trizol reagent (Life Technologies, Gaithersburg, MD, USA). Reverse transcriptase reactions were done using the PrimeScript RT reagent Kits (No. R122-01, Vazyme Biotech, Nanjing, China). RT-PCR was performed using Quantitative PCR with SYBR Premix Ex Taq™ (TaKara, Otsu, Shiga, Japan) and ABI PRISM 7500 sequence detection PCR system (Applied Biosystems, Foster City, CA, USA). The quantitative data were obtained with the *ΔΔ*CT method and normalized to GAPDH. The primer sequences for FNDC5 are listed in a supplementary material (Supplementary Table 1).

### 2.8. Measurement of SIRT1 Activity

SIRT1 activity was measured using the SIRT1 Activity Assay Kit (No. ab156065, Abcam, Cambridge, USA) following the manufacturer's protocol.

### 2.9. NLRP3 Immunofluorescence Staining

A7R5 cells were grown on glass cover slips in a 6-well plate (100,000 cells/well). The cells with a density of 80-90% were treated with PBS or FNDC5 (200 nM) for 2 h followed by PBS or Ang II (100 nM) for 24 h. The cells were washed with PBS for three times, fixed with 10% formaldehyde for 10 min, and rinsed with deionized water and permeabilized with 0.5% Triton-X-100 in PBS for 5 min. After blocking in filtered 3% bovine serum albumin for 1 h, cells were incubated with NLRP3 antibody (No. AMAB90569, 1 : 100; Sigma-Aldrich, St. Louis, MO, USA) overnight at 4°C and then incubated with secondary antibody for 1 h at room temperature. DAPI (4′6-diamidino-2-phenylindole), a blue-fluorescent DNA stain, was used for nuclear staining [45].

### 2.10. Chemicals

FNDC5 and Ang II were bought from Sigma Inc. (St. Louis, MO, USA). Compound C, GLPG0187, and EX527 were bought from MedChem Express (Greenville, SC, USA).

### 2.11. Statistical Analysis

Experimenters were blind to group assignment and outcome assessment. Comparisons between two groups were made by Student's *t*-test. One-way or two-way ANOVA was used for multiple comparisons followed by the post hoc Bonferroni's test. All data were expressed as mean ± SE. The *P* value less than 0.05 was considered statistically significant.

## 3. Results

### 3.1. FNDC5 Deficiency Promotes Ang II-Induced Hypertension and Vascular Remodeling in Mice

**Figure 1:**
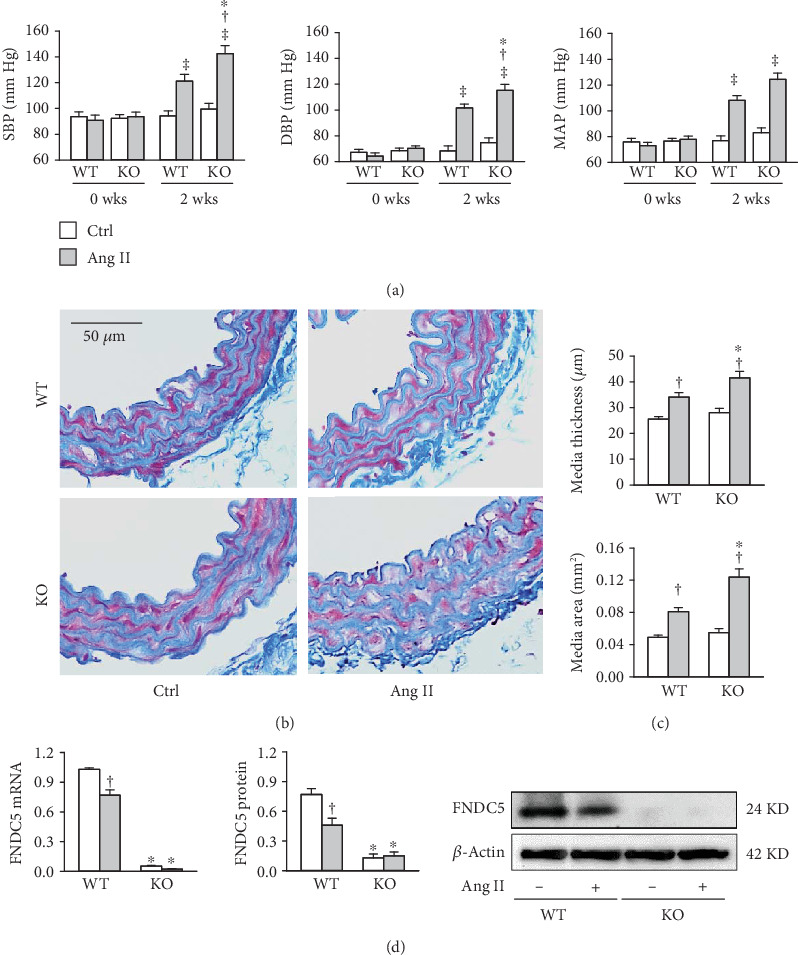
FNDC5 deficiency deteriorates Ang II-induced hypertension and vascular remodeling in mice. Ang II was infused subcutaneously with a micro-osmotic pump at 400 ng/Kg/min for 2 weeks in wild-type mice (WT) and FNDC5 knockout mice (KO). (a) Systolic blood pressure (SBP), diastolic blood pressure (DBP), and mean arterial pressure (MAP) in caudal artery were measured in awake state. (b) Representative images of Masson's staining of aorta. (c) Bar graph showing the Masson's staining analysis for media thickness and area in aorta. (d) FNDC5 mRNA and protein expressions. Values are mean ± SE. ^∗^*P* < 0.05 vs WT; ^†^*P* < 0.05 vs Ctrl; ^‡^*P* < 0.05 vs 0 wks. *n* = 6 per group.

Hypertension and vascular remodeling were induced by subcutaneous infusion of Ang II with a microosmotic pump for 2 weeks in wild-type mice (WT) and FNDC5 knockout mice (KO). PBS served as a control of Ang II. FNDC5 deficiency had no significant effects on blood pressure in the PBS-treated mice but aggravated Ang II-induced hypertension (Figure 1(a)). Ang II resulted in vascular remodeling in the aorta evidenced by increased aortic medium thickness and area in both WT and FNDC5 knockout mice, but the effects of Ang II were greater in FNDC5 knockout mice than those of WT mice (Figures 1(b) and 1(c)). On the other hand, Ang II infusion for 2 weeks reduced FNDC5 mRNA and protein expression in WT mice (Figure 1(d)).

### 3.2. FNDC5 Deficiency Aggravates Ang II-Induced Oxidative Stress and NLRP3 Inflammasome Activation in Mice

**Figure 2:**
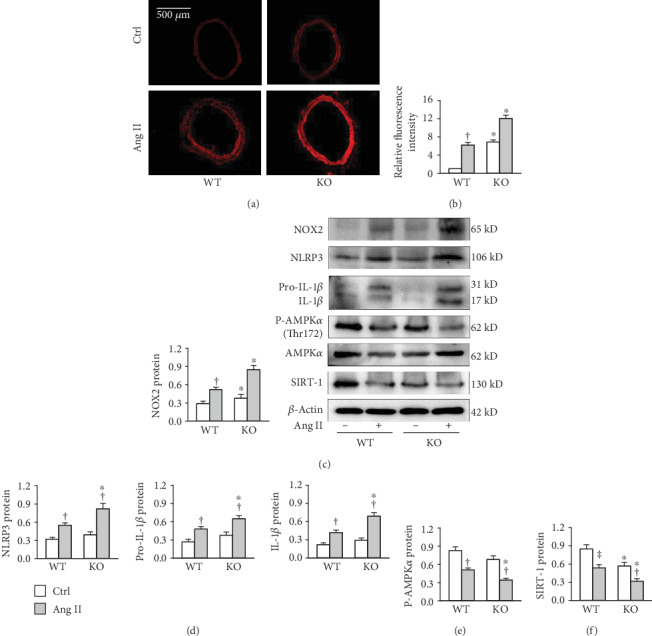
FNDC5 deficiency deteriorates Ang II-induced oxidative stress and NLRP3 inflammasome activation in aorta of mice. Ang II was infused subcutaneously with a microosmotic pump at 400 ng/Kg/min for 2 weeks. (a) Representative images showing the ROS detected by dihydroethidium (DHE) staining. (b) Bar graph showing the relative fluorescence intensity of DHE. (c) NOX2 protein expression. (d) NLRP3, pro-IL-1*β*, and IL-1*β* protein expressions. (e) Phosphorylated AMPK*α*. (f) SIRT1 protein expression. Values are mean ± SE. ^∗^*P* < 0.05 vs WT; ^†^*P* < 0.05 vs Ctrl. *n* = 4 per group.

Subcutaneous infusion of Ang II for two weeks increased ROS production and NOX2 protein expression in the aortic media of both WT and FNDC5 knockout mice. The changes were greater in FNDC5 knockout mice than those of WT mice (Figures 2(a)-2(c)). Similarly, Ang II-induced NLRP3 and pro-IL-1*β* upregulation and IL-1*β* production were amplified in FNDC5 knockout mice compared with WT mice (Figure 2(d)). SIRT1 is a NAD^+^-dependent deacetylase that is responsible for deacetylating the proteins responsible for cellular regulation. It has been found that AMPK*α*_1_ overexpression improves postoperative cognitive dysfunction via the AMPK-SIRT1 and autophagy signaling pathways [46]. Activation of SIRT1 attenuates Klotho Deficiency-induced hypertension and arterial stiffness [47]. An interesting question is whether AMPK is associated with the effects of FNDC5 on oxidative stress and inflammation. We found that Ang II-induced inhibition in the AMPK*α* phosphorylation and SIRT1 expression were intensified in FNDC5 knockout mice (Figures 2(e) and 2(f)).

### 3.3. FNDC5 Inhibits Ang II-Induced Oxidative Stress in A7R5 Cells

**Figure 3:**
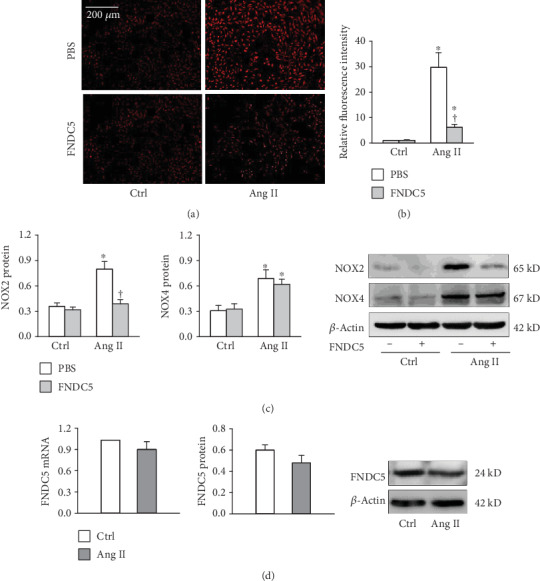
FNDC5 attenuates Ang II-induced oxidative stress in A7R5 cells. The cells were treated with FNDC5 (200 nM) for 2 h followed by Ang II (100 nM) for 24 h. (a) Representative images showing the ROS detected by dihydroethidium (DHE) staining. (b) Bar graph showing the relative fluorescence intensity of DHE. (c) NOX2 and NOX4 protein expressions. (d) FNDC5 mRNA and protein expressions. Values are mean ± SE. ^∗^*P* < 0.05 vs Ctrl; ^†^*P* < 0.05 vs PBS. *n* = 4 per group.

Ang II was utilized to induce oxidative stress and inflammation in VSMCs [30–34]. DHE fluorescence staining showed that exogenous FNDC5 inhibited Ang II-induced ROS production (Figures 3(a) and 3(b)). FNDC5 prevented Ang II-induced NOX2 upregulation but had no significant effect on Ang II-induced NOX4 upregulation (Figure 3(c)). Ang II treatment for 24 h had no significant effects on FNDC5 mRNA and protein expressions (Figure 3(d)), while Ang II treatment for 72 h reduced FNDC5 protein expression (Supplementary Figure 1).

### 3.4. FNDC5 Prevents Ang II-Induced NLRP3 Inflammasome Activation in A7R5 Cells

**Figure 4:**
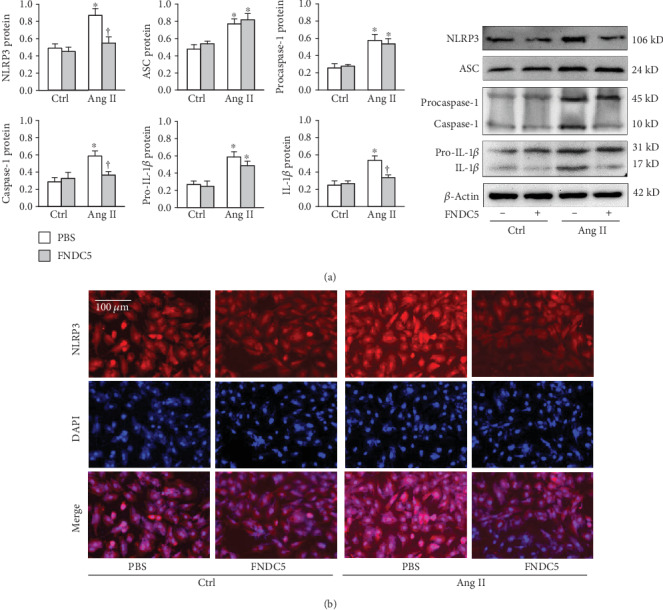
FNDC5 attenuates Ang II-induced NLRP3 inflammasome activation in A7R5 cells. The cells were treated with FNDC5 (200 nM) for 2 h followed by Ang II (100 nM) for 24 h. (a) NLRP3, ASC, procaspase-1, caspase-1, pro-IL-1*β*, and IL-1*β* protein expressions. (b) Representative images showing the immunofluorescence for NLRP3. Values are mean ± SE. ^∗^*P* < 0.05 vs Ctrl; ^†^*P* < 0.05 vs PBS. *n* = 4 per group.

FNDC5 inhibited Ang II-induced upregulation of NLRP3, caspase-1, and mature IL-1*β* but had no significant effects on Ang II-induced upregulation of ASC, procaspase-1, and pro-IL-1*β* (Figure 4(a)). These results indicate that exogenous FNDC5 inhibits Ang II-induced NLRP3 inflammasome activation, which may be related to its downregulation effect on NLRP3. The findings were further confirmed by immunofluorescence data that FNDC5 prevented the Ang II-induced NLRP3 expression (Figure 4(b)).

### 3.5. Inhibition of AMPK Prevents the Effects of FNDC5 in A7R5 Cells

**Figure 5:**
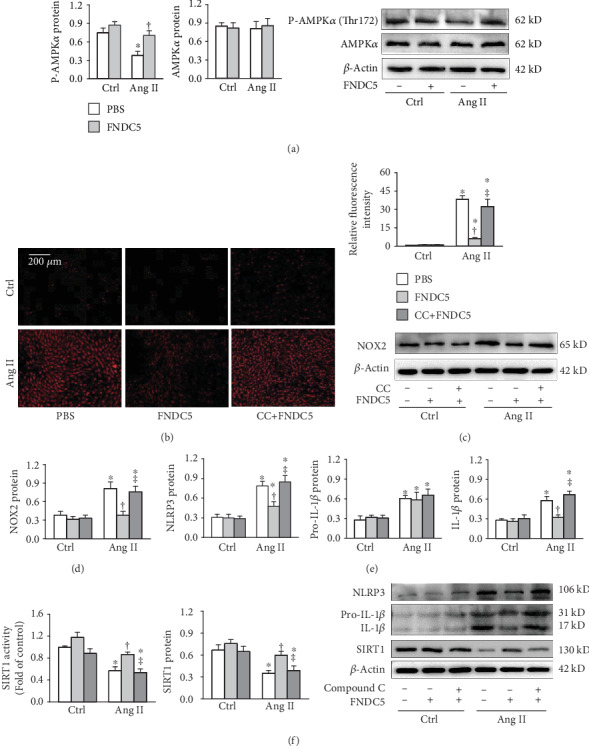
Inhibition of AMPK with compound C abolishes the protective effects of FNDC5 in Ang II-treated A7R5 cells. The cells were treated with combined compound C (20 *μ*M) and FNDC5 (200 nM) for 2 h followed by Ang II (100 nM) for 24 h. (a) Phosphorylated AMPK*α* and AMPK*α* protein expression. (b) Representative images showing the ROS detected by dihydroethidium (DHE) staining. (c) Bar graph showing the relative fluorescence intensity of DHE. (d) NOX2 expression. (e) NLRP3, pro-IL-1*β*, and IL-1*β* protein expressions. (f) SIRT1 activity and SIRT1 protein expression. Values are mean ± SE. ^∗^*P* < 0.05 vs Ctrl; ^†^*P* < 0.05 vs PBS. ^‡^*P* < 0.05 vs FNDC5. *n* = 4 per group.

FNDC5 had no significant effect on AMPK*α* protein expression but prevented the Ang II-induced AMPK*α* phosphorylation inhibition in A7R5 cells (Figure 5(a)). Compound C, a cell-permeable AMPK inhibitor, attenuated the roles of FNDC5 in inhibiting the Ang II-induced ROS production (Figures 5(b) and 5(c)), NOX2 upregulation (Figure 5(d)), and NLRP3 and mature IL-1*β* upregulation (Figure 5(e)) in A7R5 cells. These results indicate that FNDC5 attenuates Ang II-induced oxidative stress and inflammation by restoring the AMPK*α* phosphorylation. Furthermore, Ang II inhibited SIRT1 activity and protein expression which were attenuated by FNDC5, and the effects of FNDC5 were further abolished by the treatment with compound C (Figure 5(f)).

### 3.6. Inhibition of SIRT1 Abolishes the Effects of FNDC5 in A7R5 Cells

**Figure 6:**
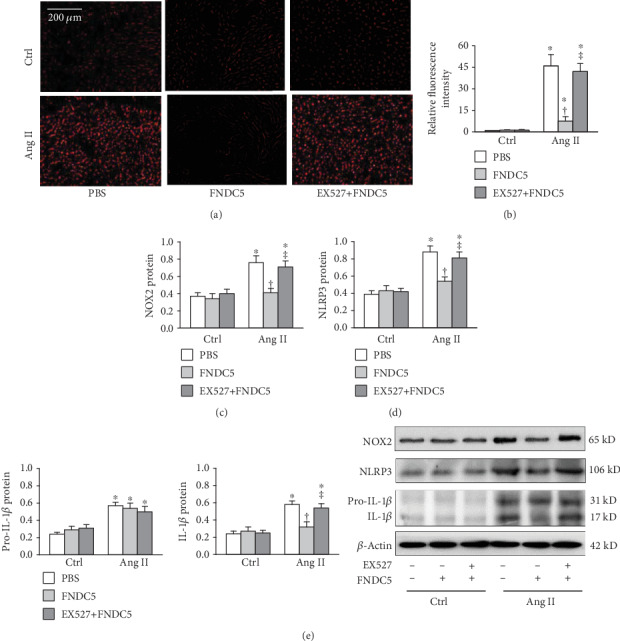
Inhibition of SIRT-1 with EX527 attenuates the protective effects of FNDC5 in Ang II-treated A7R5 cells. The cells were treated with combined EX527 (25 *μ*M) and FNDC5 (200 nM) for 2 h followed by Ang II (100 nM) for 24 h. (a) representative images showing the ROS detected by dihydroethidium (DHE) staining. (b) Bar graph showing the relative fluorescence intensity of DHE. (c) NOX2 expression. (d) NLRP3 protein expression. (e), Pro-IL-1*β* and IL-1*β* expressions. Values are mean ± SE. ^∗^*P* < 0.05 vs Ctrl; ^†^*P* < 0.05 vs PBS. ^‡^*P* < 0.05 vs FNDC5. *n* = 4 per group.

EX527, a selective inhibitor of SIRT1, abolished the roles of FNDC5 in inhibiting the Ang II-induced ROS production (Figures 6(a) and 6(b)), NOX2 upregulation (Figure 6(c)), and NLRP3 and mature IL-1*β* upregulation (Figures 6(d) and 6(e)) in A7R5 cells. These results indicate that FNDC5 attenuates Ang II-induced oxidative stress and inflammation via the AMPK*α*-SIRT1 pathway.

### 3.7. Inhibition of Integrins Prevents the Effects of FNDC5 in A7R5 Cells

**Figure 7:**
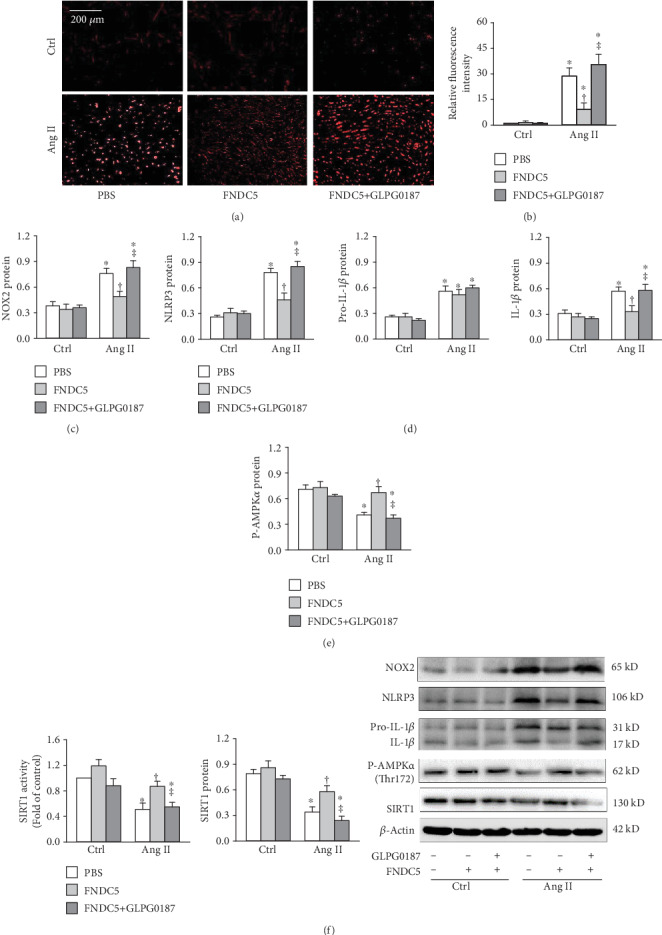
Inhibition of integrin receptor with GLPG0187 attenuates the protective effects of FNDC5 in Ang II-treated A7R5 cells. The cells were treated with GLPG0187 (1 nM) and FNDC5 (200 nM) for 2 h followed by Ang II (100 nM) for 24 h. (a) Representative images showing the ROS detected by dihydroethidium (DHE) staining. (b) Bar graph showing the relative fluorescence intensity of DHE. (c) NOX2 expression. (d) NLRP3, pro-IL-1*β*, and IL-1*β* protein expressions. (e) Phosphorylated AMPK*α*. (f) SIRT1 activity and SIRT1 protein expression. Values are mean ± SE. ^∗^*P* < 0.05 vs Ctrl; ^†^*P* < 0.05 vs PBS. ^‡^*P* < 0.05 vs FNDC5. *n* = 4 per group.

Integrins are heterodimeric cell surface adhesion receptors that are involved in activating intracellular signaling pathways associated with cell proliferation, adhesion, migration, spreading, differentiation, and survival [48]. Integrins play a critical role in eliciting a protective response to oxidative damage in epidermal cells [49] and are essential for leukocyte adhesion and migration in various inflammatory diseases [50]. Recently, it has been found that integrins are the receptors of irisin in adipose tissues and osteocytes [51]. We suspect that integrins might be involved in the effects of FNDC5. Thus, GLPG0187, a broad spectrum integrin receptor antagonist [52], was used to determine whether integrins were mediated the effects of FNDC5. Just as expected, GLPG0187 prevented the roles of FNDC5 in attenuating the Ang II-induced upregulation of NOX2 and NLRP3 and the production of the ROS and IL-1*β* in A7R5 cells (Figures 7(a)-7(d)). Furthermore, GLPG0187 abolished the roles of FNDC5 in attenuating the Ang II-induced inhibition in AMPK*α* phosphorylation and SIRT1 activity and expression in A7R5 cells (Figures 7(e)-7(f)). Inhibition of AMPK, SIRT-1, or integrin receptors had no significant effects on FNDC5 expression in Ang II-treated A7R5 cells (Supplementary Figure 2).

### 3.8. FNDC5 Prevents Ang II-Induced Oxidative Stress and NLRP3 Inflammasome Activation in Primary VSMCs of WT and FNDC5 Knockout Mice

**Figure 8:**
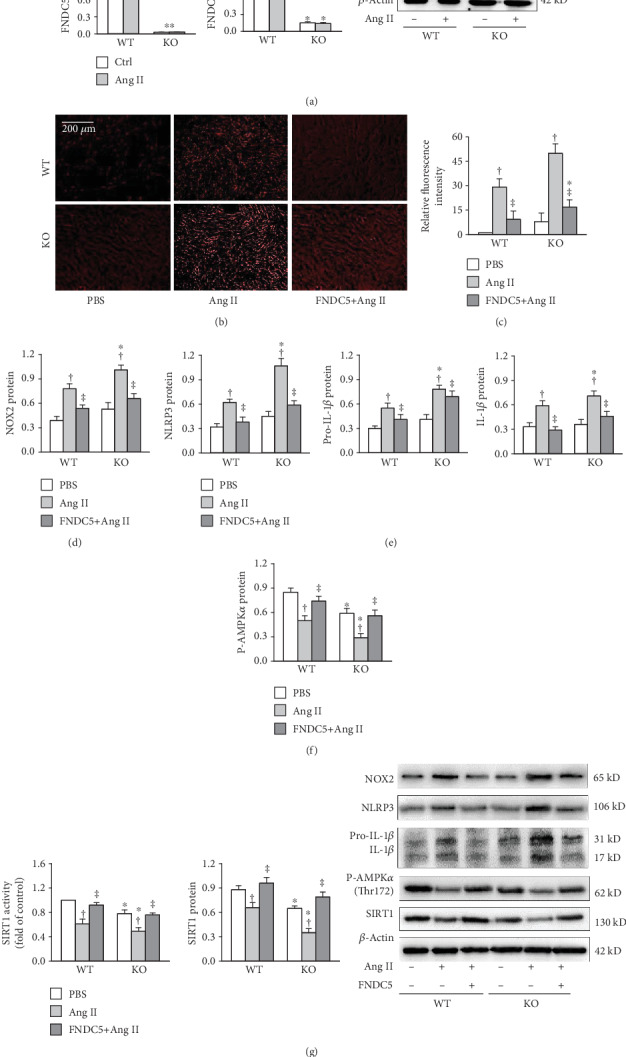
FNDC5 prevents the enhanced oxidative stress and NLRP3 inflammasome activation in Ang II-treated VSMCs of FNDC5^−/−^ mice. The primary VSMCs of the WT and FNDC5 knockout (KO) mice were treated with PBS or FNDC5 (200 nM) for 2 h followed by PBS or Ang II (100 nM) for 24 h. (a) FNDC5 mRNA and protein expressions. (b) Representative images showing the ROS detected by dihydroethidium (DHE) staining. (c) Bar graph showing the relative fluorescence intensity of DHE. (d) NOX2 protein expression. (e) NLRP3, pro-IL-1*β*, and IL-1*β* protein expressions. (f) Phosphorylated AMPK*α*. (g) SIRT1 activity and SIRT1 protein expression. Values are mean ± SE. ^∗^*P* < 0.05 vs WT; ^†^*P* < 0.05 vs PBS or Ctrl. ^‡^*P* < 0.05 vs Ang II. *n* = 4 per group.

Ang II had no significant effect on FNDC5 mRNA and protein expressions in the primary VSMCs of WT and FNDC5 knockout mice (Figure 8(a)). Exogenous FNDC5 attenuated Ang II-induced ROS production and NOX2 upregulation in VSMCs of both WT and FNDC5 knockout mice (Figures 8(b) and 8(d)). It also prevented the Ang II-induced NLRP3 upregulation and IL-1*β* production in the VSMCs (Figure 8(e)). Moreover, Ang II-induced inhibition in AMPK*α* phosphorylation, SIRT1 activity, and expression were attenuated by FNDC5 treatment (Figures 8(f) and 8(g)).

## 4. Discussion

Vascular oxidative stress and inflammation are closely related with vascular remodeling in cardiovascular diseases such as hypertension, atherosclerosis, vascular restenosis, and diabetic vascular complications [7]. Intervention of vascular oxidative stress and inflammation attenuate vascular remodeling in these diseases [11, 13]. Our previous studies showed the beneficial roles of FNDC5 in attenuating the disturbance of glucose and lipid metabolism, insulin resistance, and hepatosteatosis [24–26]. Recently, we found that FNDC5 reduced NOX2-derived ROS production, NLRP3 inflammasome activation, and phenotypic transformation in the adventitial fibroblasts of SHR [28]. VSMCs are the dominant cellular constituent of arteries and play critical roles in vascular remodeling. Ang II induces oxidative stress, inflammation, proliferation, and migration of VSMCs and greatly contributes to vascular remodeling in hypertension and other vascular diseases [14, 53]. Based on the importance of VSMCs and Ang II in vascular remodeling, it is very important to explore whether FNDC5 has a protective role in attenuating Ang II-induced oxidative stress and inflammation in VSMCs. The primary novel findings in the present study are that FNDC5 deficiency aggravates Ang II-induced hypertension, vascular oxidative stress, NLRP3 inflammasome activation, and vascular remodeling in mice, and that exogenous FNDC5 alleviates the Ang II-induced oxidative stress and NLRP3 inflammasome activation in VSMCs. These results suggest that FNDC5 might be a promising therapeutical strategy in attenuating vascular oxidase stress and inflammation in vascular diseases.

Ang II increases NOX activity, ROS production, and inflammation mediated by AT_1_ receptors [54, 55]. It inhibits AMPK activation in VSMCs of SHR [56]. AMPK*α*_1_ overexpression increased the phosphorylated AMPK and SIRT1 expressions in the hippocampus of rats [46]. In the present study, Ang II inhibited AMPK*α* phosphorylation and SIRT1 expression and activity, which were prevented by FNDC5. Inhibition of AMPK or SIRT1 prevented the beneficial roles of FNDC5 in attenuating Ang II-induced NOX2 and NLRP3 upregulation and ROS production but had no significant effects on FNDC5 expression. Furthermore, inhibition of AMPK abolished the roles of FNDC5 in preventing Ang II-induced SIRT1 downregulation. These findings suggest that the roles of FNDC5 in attenuating vascular oxidative stress and NLRP3 inflammasome activation are mediated by the AMPK-SIRT1 pathway. It is noted that FNDC5 prevented Ang II-increased NOX2 upregulation rather than NOX4 upregulation, suggesting that the role of FNDC5 in attenuating ROS production is mediated by inhibiting Ang II-increased NOX2 upregulation. It is known that high blood pressure contributes to oxidative stress and inflammation. In the present study, Ang II treatment caused more severe hypertension, vascular remodeling, oxidative stress, and inflammasome activation in FNDC5 knockout mice than those in WT mice. It is probably that FNDC5 deficiency aggravates Ang II-induced oxidative stress and inflammasome activation, and Ang II-induced hypertension also promotes oxidative stress and inflammasome activation. On the other hand, the enhanced oxidative stress and inflammasome activation in FNDC5 deficiency mice exacerbates hypertension and vascular remodeling.

Recently, it has been found that inhibition of integrins blocks signaling and function of irisin, a cleaved peptide from FNDC5, in osteocytes and fat cells [51]. We found that inhibition of integrins with GLPG0187, a broad inhibitor of integrin family receptors, abolished the protective roles of FNDC5 in Ang II-induced AMPK-SIRT1 inhibition, oxidative stress, and inflammation but had no significant effects on FNDC5 expression. The findings suggest that the effects of FNDC5 are mediated by integrins, which was supported by the findings that inhibition of integrins blocks signaling and function of irisin, a cleaved peptide from FNDC5, in osteocytes and fat cells [51], and that AMPK mediates the roles of FNDC5 in attenuating adipose tissue inflammation [27]. However, it is unknown whether the effects of FNDC5 are caused directly by acting on integrins or indirectly by its cleaved peptide irisin acting on the integrins, which is a limitation in the present study. On the other hand, Ang II treatment for 4 h and 24 h in VSMCs had no significant effects on FNDC5 expressions, while Ang II treatment for 72 h in VSMCs or Ang II infusion for 2 weeks in mice significantly reduced the aortic FNDC5 expressions. These results suggest that Ang II has no direct inhibitory effect on FNDC5 expressions in VSMCs, and the downregulation of FNDC5 in the sustained Ang II-treated VSMCs or Ang II-infused mice may be caused by its secondary effects.

## 5. Conclusions

FNDC5 deficiency exacerbates oxidative stress and NLRP3 inflammasome activation in VSMCs, while exogenous FNDC5 alleviates the Ang II-induced oxidative stress and NLRP3 inflammasome activation in VSMCs. Integrin-mediated AMPK-SIRT1 activation is involved in the protective effects of FNDC5 on vascular oxidative stress and NLRP3 inflammasome activation.

## Data Availability

The raw data supporting the findings of this study are available from the corresponding author on reasonable request.

## Supplementary Materials

Supplementary Materials Supplementary Figure 1: effects of Ang II on FNDC5 protein expressions in A7R5 cells. The cells were treated with Ang II (100 nM) for 4 h or 72 h. Values are mean ± SE. ^∗^*P* < 0.05 vs Ctrl. *n* = 3 per group. Supplementary Figure 2: effects of compound C (an AMPK inhibitor), EX527 (a SIRT-1 inhibitor), and GLPG0187 (an integrin receptor inhibitor) on FNDC5 protein expressions in Ang II-treated A7R5 cells. The cells were treated with compound C (20 *μ*M), EX527 (25 *μ*M), or GLPG0187 (1 nM) for 2 h followed by Ang II (100 nM) for 24 h. Values are mean ± SE. No significant difference was found among groups. *n* = 3 per group. Supplementary Table 1: primers for real-time quantitative PCR analysis.
