# Supplementary material for: FNDC5 Attenuates Oxidative Stress and NLRP3 Inflammasome Activation in Vascular Smooth Muscle Cells via Activating the AMPK-SIRT1 Signal Pathway
Source: Oxid Med Cell Longev. 2020 May 16;2020:6384803. doi: 10.1155/2020/6384803 (PMC7254086; doi:10.1155/2020/6384803)
Supplement: Supplementary Materials — Supplementary Figure 1: effects of Ang II on FNDC5 protein expressions in A7R5 cells. The cells were treated with Ang II (100 nM) for 4 h or 72 h. Values are mean ± SE. ∗P < 0.05 vs Ctrl. n = 3 per group. Supplementary Figure 2: effects of compound C (an AMPK inhibitor), EX527 (a SIRT-1 inhibitor), and GLPG0187 (an integrin receptor inhibitor) on FNDC5 protein expressions in Ang II-treated A7R5 cells. The cells were treated with compound C (20 μM), EX527 (25 μM), or GLPG0187 (1 nM) for 2 h followed by Ang II (100 nM) for 24 h. Values are mean ± SE. No significant difference was found among groups. n = 3 per group. Supplementary Table 1: primers for real-time quantitative PCR analysis. [file 6384803.f1.pdf]

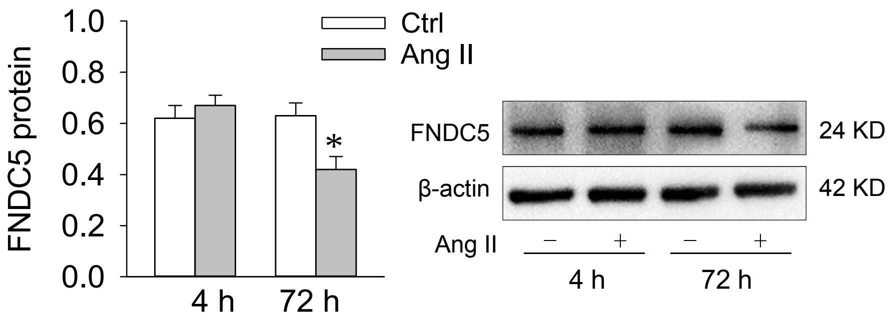

Supplementary Figure 1. Effects of Ang II on FNDC5 protein expressions in A7R5 cells. The cells were treated with Ang II (100 nM) for 4 h or 72 h. Values are mean±SE. *$P<0.05$ vs Ctrl. n=3 per group.

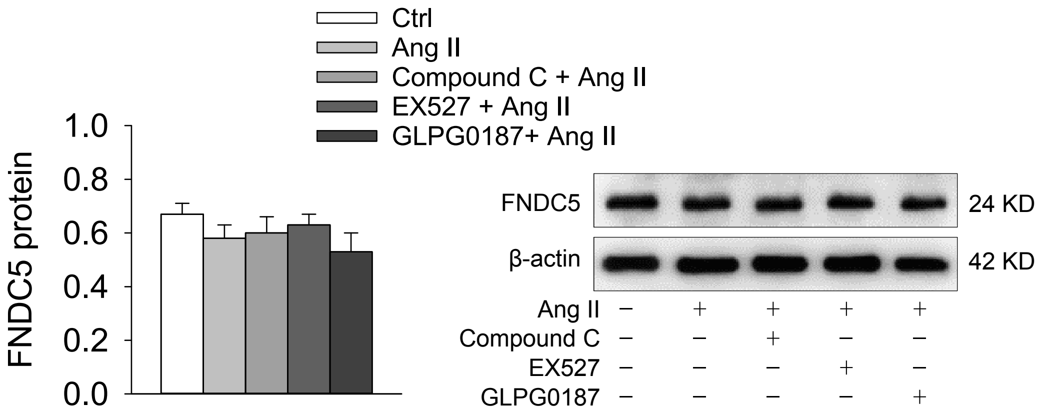

Supplementary Figure 2. Effects of compound C (an AMPK inhibitor), EX527 (a SIRT-1 inhibitor), GLPG0187 (an integrin receptor inhibitor) on FNDC5 protein expressions in Ang II-treated A7R5 cells. The cells were treated with compound C (20 μM), EX527 (25 μM) or GLPG0187 (1 nM) for 2 h followed by Ang II (100 nM) for 24 h. Values are mean±SE. No significant difference was found among groups. n=3 per group.

Supplementary Table 1 Primers for real-time quantitative PCR analysis

Animal	Gene	Primer	Sequence
Mouse	FNDC5	Forward	AGCCAGCAGGAGTTTCTATG’
		Reverse	GCGACAGCATTTGGACTCAA
	GAPDH	Forward	AGGTTGTCTCCTGCGACTTCA
		Reverse	TGGTCCAGGGTT TCTTACTCC
Rat	FNDC5	Forward	TCATTGTTGTGGTCCTCTTC-3′
		Reverse	GCTCGTTGTCCTTGATGATA
	GAPDH	Forward	TTCTTGTGCAGTGCCAGCCTCGTC
		Reverse	TAGGAACACGGAAGGCCATGCCAG
